# Regulatory T cells suppress the motility of cytotoxic T cells in Friend retrovirus–infected mice

**DOI:** 10.1172/jci.insight.167482

**Published:** 2023-07-10

**Authors:** Daniela Mittermüller, Lucas Otto, Zoë Long, Andreas Kraus, Alexander Beer, Anja Hasenberg, Gennadiy Zelinskyy, Jaana Westmeier, Kim J. Hasenkrug, Ulf Dittmer, Matthias Gunzer

**Affiliations:** 1 Institute for Virology and; 2 Institute for Experimental Immunology and Imaging, University Hospital Essen, University of Duisburg-Essen, Essen, Germany.; 3 Rocky Mountain Laboratories, National Institute of Allergy and Infectious Diseases, NIH, Hamilton, Montana, USA.; 4 Institute for Translational HIV Research, University Hospital Essen, University of Duisburg-Essen, Essen, Germany.; 5 Leibniz-Institute for Analytical Sciences ISAS-e.V., Dortmund, Germany.

**Keywords:** Immunology, Virology, Adaptive immunity, Cell migration/adhesion, T cells

## Abstract

Antiviral immunity often requires CD8^+^ cytotoxic T lymphocytes (CTLs) that actively migrate and search for virus-infected targets. Regulatory T cells (Tregs) have been shown to suppress CTL responses, but it is not known whether this is also mediated by effects on CTL motility. Here, we used intravital 2-photon microscopy in the Friend retrovirus (FV) mouse model to define the impact of Tregs on CTL motility throughout the course of acute infection. Virus-specific CTLs were very motile and had frequent short contacts with target cells at their peak cytotoxic activity. However, when Tregs were activated and expanded in late-acute FV infection, CTLs became significantly less motile and contacts with target cells were prolonged. This phenotype was associated with development of functional CTL exhaustion. Tregs had direct contacts with CTLs in vivo and, importantly, their experimental depletion restored CTL motility. Our findings identify an effect of Tregs on CTL motility as part of their mechanism of functional impairment in chronic viral infections. Future studies must address the underlying molecular mechanisms.

## Graphical abstract

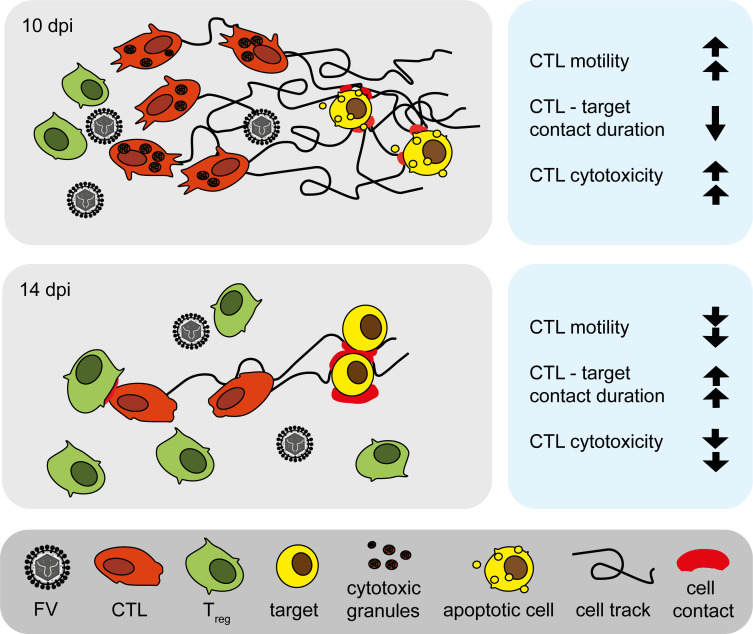

## Introduction

Cytotoxic T lymphocytes (CTLs) are key components of the host immune response against viral infections, including those with retroviruses (1–4). They efficiently eliminate virus-infected cells through the release of cytotoxic molecules, like granzymes and perforin, via exocytosis (2, 5–8). In order to deliver these lytic granules, CTLs directly contact virus-infected cells and form immunological synapses and kinapses (6, 9). To achieve serial contacts with several virus-infected cells, CTLs need to actively migrate and search for target cells (5). CTL motility has therefore been associated with the efficiency of target cell elimination in several disease models, including infections and cancers (5, 10, 11).

In the course of an ongoing immune activation, CTLs can become impaired in their antiviral response. This phenomenon is called T cell exhaustion and contributes to incomplete virus elimination and the development of chronic infections (12, 13). To date, T cell exhaustion has been widely characterized based on changes in the molecular expression profile of T cells, where research prominently focused on the decrease in cytotoxic molecule expression and the increase in immune checkpoint molecules (14, 15). Today, T cell exhaustion is a highly relevant topic, as it impairs effective immune responses in a multitude of diseases, including chronic hepatitis C and HIV infections, as well as many cancers (14). Reactivation of exhausted CTLs is a valuable therapeutic goal and remains a focus of research, but many gaps in the understanding of the mechanisms of T cell exhaustion remain, especially in infectious diseases.

Deficient CTL responses due to T cell exhaustion contribute to the development of chronic Friend virus (FV) infections in mice (12, 16). FV is a murine retrovirus model system. Similar to HIV in humans, FV infection in C57BL/6 mice is controlled during acute infection, but the virus is not completely eliminated, resulting in life-long viral chronicity. Studies in FV paved the way for better understanding of the immune response against acute and chronic retrovirus infections, which has made FV a valuable tool to investigate the mechanisms of T cell exhaustion (16–18).

When regulatory T cells (Tregs) were discovered in the late 1990s, their role in infectious diseases was largely unexplored. Research utilizing the FV mouse model significantly contributed to our current knowledge that Tregs are important players in many infectious diseases by counter-regulating CTL responses to prevent immunopathology during acute infections (17, 19). On the other hand, Treg activity can prevent complete elimination of viruses by CTLs and thus contribute to the development of chronic infections (12, 19). Consequently, therapeutic depletion of Tregs during persistent FV infection can reactivate exhausted CTLs and reduce infection levels (12). In these studies, DEREG mice were used in which Tregs express both EGFP and the primate diphtheria toxin receptor. Thus, Tregs can be visualized by microscopy and selectively depleted by injecting diphtheria toxin (20). The first functional interactions of Tregs and CTLs take place during the acute and late-acute phase of FV infection in mice (15). In kinetic studies using flow cytometry, we were able to define 3 important time points during acute FV infection that are also subject to study in our current experiments: (i) 8 days post infection (dpi) is the time point when detectable anti-FV CTL activity starts, but only very few Tregs can be found; (ii) 10 dpi: peak of the antiviral CTL response, whereas the Treg response has just started to develop; (iii) 14 dpi: strong Treg activation, which starts to dampen the CTL response (8). These phenomena can be studied best in the bone marrow of infected mice, because no virus-specific CTLs and almost no Tregs are present in the bone marrow of naive mice (15). However, after FV infection, extensive viral replication takes place in the bone marrow, because FV preferentially infects erythroid precursor cells, myeloid cells, and B cells (21). Consequently, FV-specific CTLs infiltrate the bone marrow in order to kill infected target cells, followed thereafter by Tregs that start to suppress CTL functions (15). The aim of this study was therefore to visualize these important cellular players of antiviral immunity and their movement and interaction under physiological conditions in the living mouse.

Although studies in chronic lymphocytic choriomeningitis virus (LCMV) infection highlighted the impact of T cell exhaustion on the motility of T cells (10), little is currently known about CTL migration throughout the acute and chronic phases of retroviral infections and the impact of Treg responses on these parameters. A powerful tool to study the motility of immune cells in vivo is intravital 2-photon microscopy (22, 23). We established this technique to image leukocytes in the marrow of long bones (24). Here, we have adapted the technology to study virus-specific CTLs in FV-infected mice to evaluate CTL motility, the efficiency of target elimination in vivo, and the impact of Tregs. The visualization of this complex interplay in its natural microenvironment advances our understanding of CTL exhaustion and the interaction of effector T cells and Tregs in the immune response against chronic viral infections.

## Results

### Intravital bone marrow imaging highlights changes in CTL motility during the course of acute FV infection.

**Figure 1:**
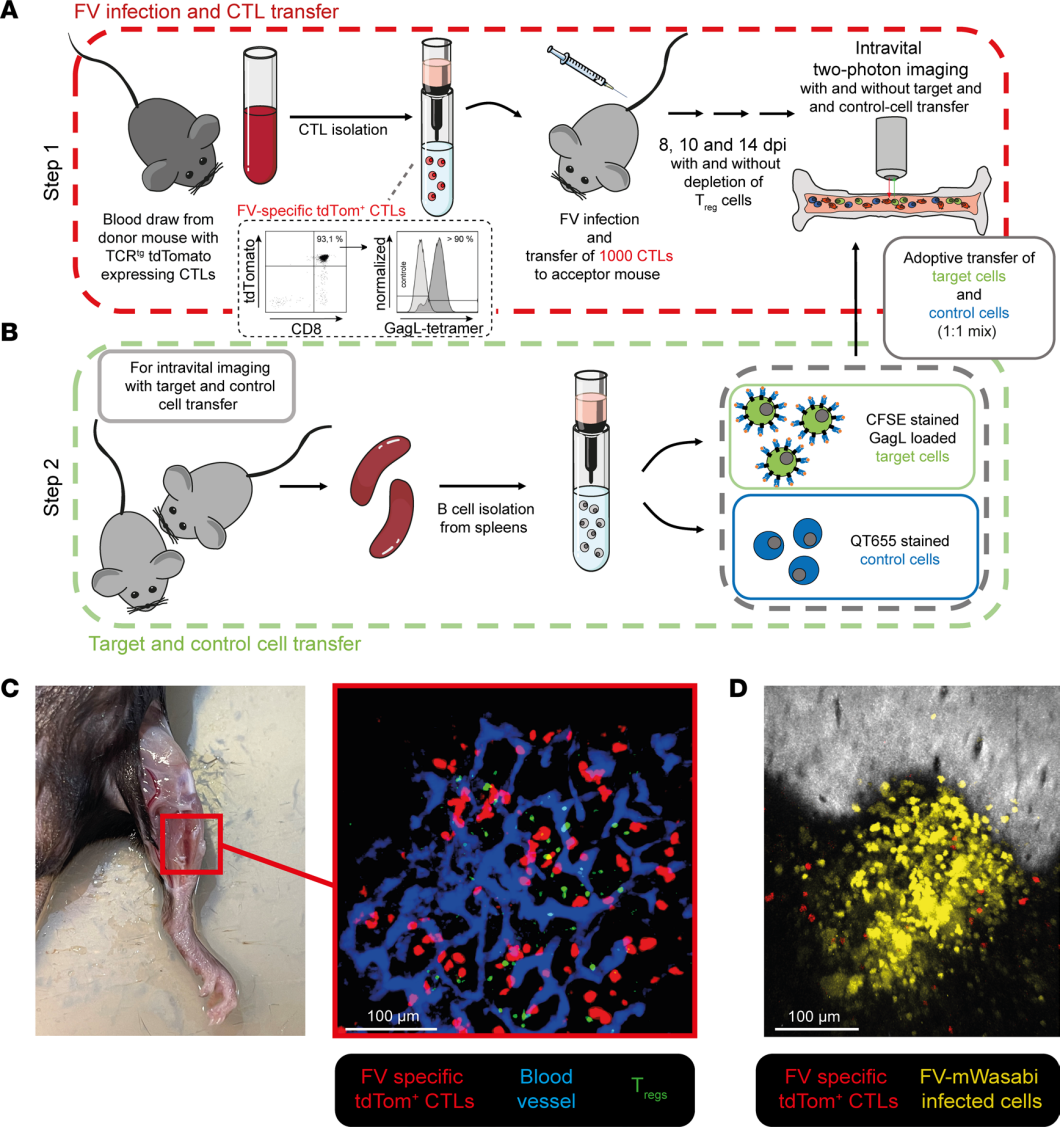
Evaluation of CTL motility through intravital 2-photon imaging. (**A** and **B**) Schematic representation of the experimental design of the intravital 2-photon bone marrow imaging. (**A**) C57BL/6 or DEREG acceptor mice were infected with FV followed by the transfer of 1000 FV-specific tdTom^+^ CTLs, obtained by blood draw from TCR-Lck-tdTom donor mice. Intravital 2-photon bone marrow imaging was performed on 8, 10, and 14 dpi. For visualization or additional depletion of Tregs, DEREG mice were used as acceptor mice and treated with diphtheria toxin or left untreated. (**B**) For evaluation of contacts with potential targets, a mix of target and control cells prepared from B cells isolated from the spleens of donor mice were transferred to FV-infected, CTL-transferred C57BL/6 or DEREG (Treg depletion) recipient mice directly before imaging at 8, 10, and 14 dpi. Tregs were depleted in FV-infected, CTL-transferred DEREG mice for visualization 14 dpi in the absence of Tregs. (**C**) Image of a mouse that underwent surgery for intravital bone marrow imaging and exemplary picture of the bone marrow obtained by intravital 2-photon microscopy. Red: FV-specific tdTom^+^ CTLs. Blue: blood flow was visualized using QT655. Green: EGFP^+^ Tregs. (**D**) Representative tibial image obtained from FV-mWasabi–infected CTL-transferred mouse 8 dpi in intravital 2-photon microscopy (yellow: FV-infected cells; red: FV-specific tdTom^+^ CTLs). Scale bars: 100 μm.

**Figure 2:**
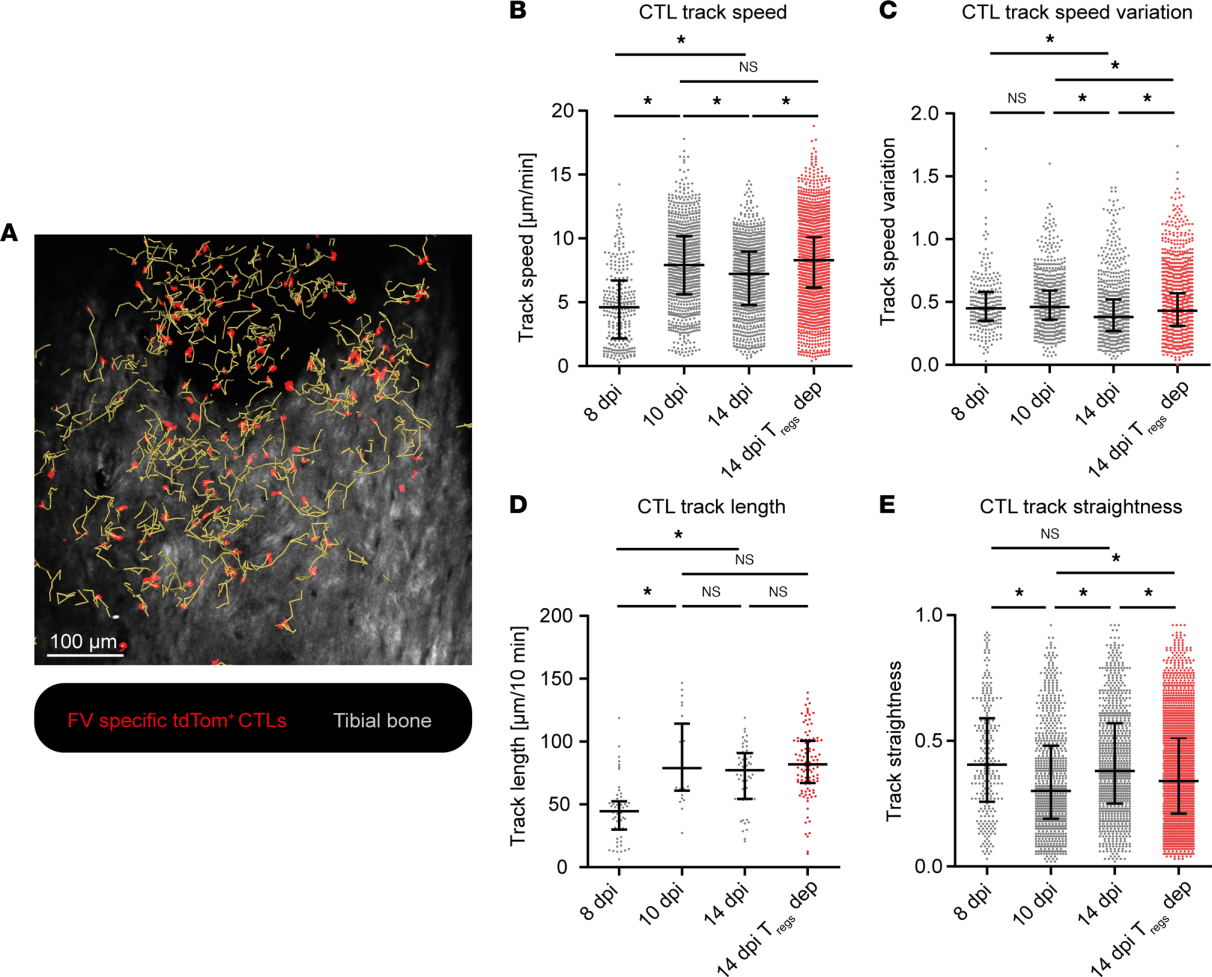
Changes in CTL motility in the course of FV infection. Intravital 2-photon microscopy in the bone marrow: CTLs were tracked in FV-infected, CTL–transferred DEREG mice 8, 10, and 14 dpi with and without Treg depletion to assess CTL motility. (**A**) Exemplary image of the CTL tracks analyzed for different CTL motility aspects. Red: CTLs. Yellow tracks: generated CTL tracks. Gray: tibial bone was visualized through the second harmonic generation signal. Scale bar: 100 μm. For the full video, see Supplemental Video 1. (**B**) CTL track speed (μm/min). (**C**) Track speed variation of single CTLs. (**D**) CTL track length in 10 minutes (μm). (**E**) Track straightness analyzed from individual CTL tracks. Data represent the values of single cells in 3 individual mice per group (median ± IQR). *P* values were obtained by Kruskal-Wallis test followed by Dunn’s multiple-comparison test. *P < 0.05.

As a first step of our approach, we evaluated the motility of CTLs and Tregs during acute FV infection by intravital bone marrow imaging in FV-infected DEREG mice, as described by Otto et al. (22). FV GagL–specific TCR-transgenic CD8^+^ T cells (1 × 10^3^) that expressed tdTomato (tdTom) were adoptively transferred into acceptor mice, which were infected with FV approximately 4 hours prior to the cell transfer (Figure 1A). We transferred only this low number of T cells because we previously showed that this did not change the course of FV infection (22). However, throughout the course of infection the transferred cells expanded sufficiently (~5 × 10^5^ CTLs on day 10) to provide enough visible CTLs to perform the analysis (22). It needs to be emphasized that FV-infected mice activate their own FV-specific CTLs in the course of their adaptive immune response, which were not directly observable because they do not express fluorescent proteins. Our generated motility and contact data are, therefore, based only on the fraction of transferred FV GagL–specific TCR-transgenic tdTom^+^ CTLs in the bone marrow. We chose the tibial bone marrow because it is an important site of active virus replication during the acute phase of FV infection (Figure 1D), and prominent expansion of CTLs and subsequently Tregs can be observed here (15, 16). This allowed us to image T cells over time in their natural environment during an acute antiviral immune response. At 8 dpi (low CTL activity, no Treg activity), 10 dpi (high CTL activity, low Treg activity), and 14 dpi (reduced CTL activity, high Treg activity) (16, 19), we performed bone marrow surgery prior to intravital 2-photon imaging (Figure 1C) (24) and investigated the CTL motility by tracking single-cell movement with an automated tool (Figure 2A and Supplemental Video 1; supplemental material available online with this article; https://doi.org/10.1172/jci.insight.167482DS1). In order to investigate CTL motility in the absence of Tregs, we used diphtheria toxin to deplete Tregs from DEREG mice (20). Motility parameters that were investigated included CTL track speed, track speed variation (standard deviation of the speed of single CTLs), track length, and track straightness (track length divided by the direct track distance).

At 8 days after FV infection, when CTL activity was still low (8, 15), the motility of CTLs in the bone marrow was very moderate. CTLs showed a median track speed of less than 5 μm/min, resulting in a median track length of approximately 45 μm in 10 minutes (Figure 2, B and D) and high track speed variation and track straightness (Figure 2, C and E). At 10 dpi, when FV-specific CTL responses peaked in our previous studies (8, 15), we observed a much stronger CTL motility characterized by significantly increased track speed and length (Figure 2, B and D) as well as reduced track straightness (Figure 2E). The latter suggested an increased CTL local search pattern, probably in search of infected targets for lysis. Interestingly, in the late-acute phase at 14 days after FV infection, when Tregs are known to initiate the suppression of CTL functions (15), we observed a significant reduction in CTL motility, documented by reduced track speeds and track speed variation (Figure 2, B and C), and again an increased track straightness (Figure 2E) compared with 10 dpi.

To clarify whether this reduction in CTL motility and change in their search pattern was indeed mediated by Tregs, we injected DEREG mice with diphtheria toxin to deplete their Tregs at 14 dpi. Indeed, Treg removal restored CTL motility to track speeds (Figure 2B) very similar to what was seen on day 10. In addition, the track speed variation was increased (Figure 2C) and track straightness was again reduced (Figure 2E). Taken together, these results show that CTL movements at 14 dpi were restored to day 10 values following Treg depletion. This result indicated that CTL motility correlates with their functional properties (8, 15) and suggest that Tregs can contribute to functional suppression of CTLs by modulating their motility during the late-acute phase of FV infection in vivo.

### Tregs show less motility than CTLs.

**Figure 3:**
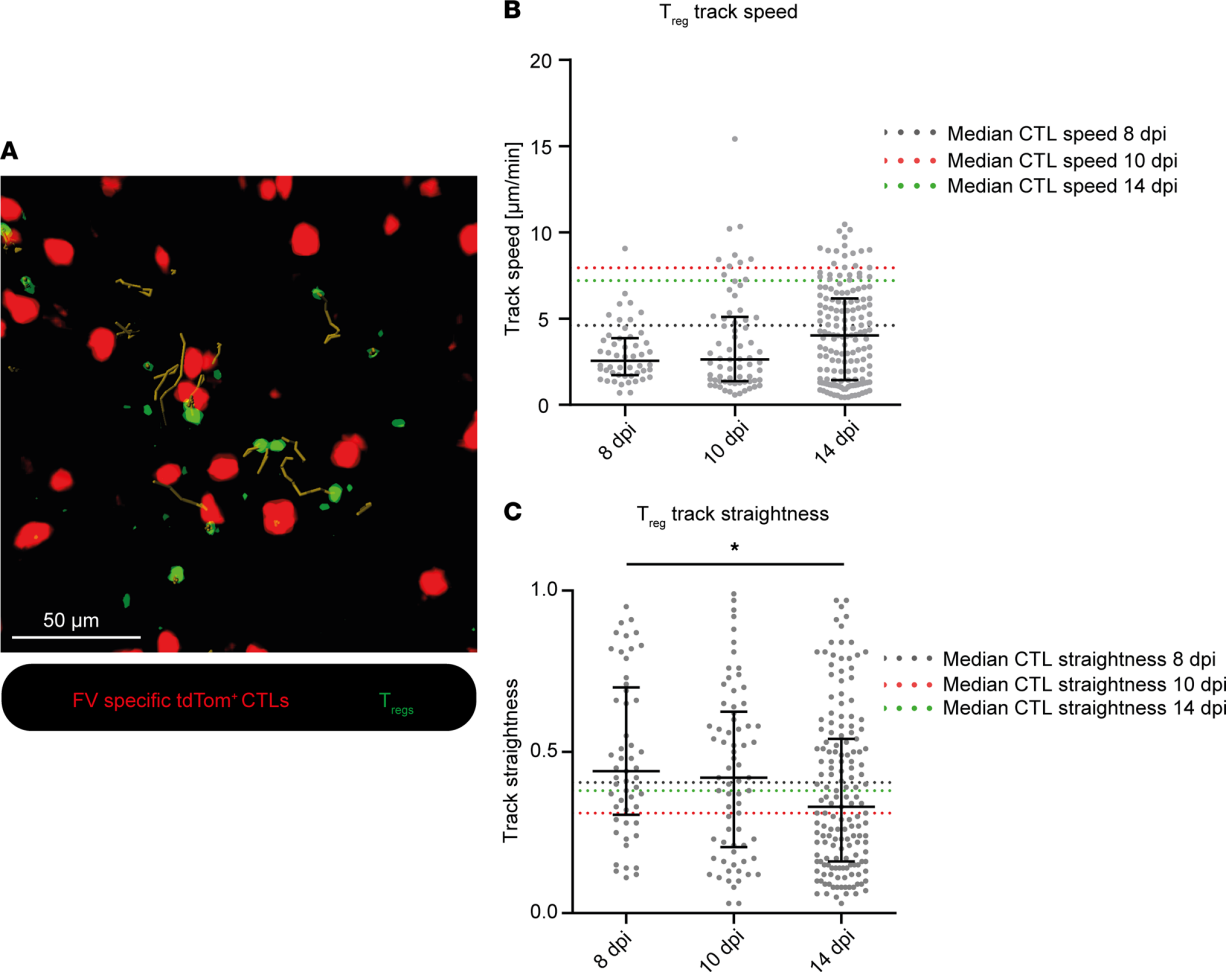
Changes in Treg motility in the course of FV infection. Intravital 2-photon microscopy in the bone marrow: Tregs were tracked in FV-infected, CTL-transferred DEREG mice 8, 10, and 14 dpi without Treg depletion to assess Treg motility. (**A**) Exemplary image of the Treg tracks. Red: FV-specific tdTom^+^ CTLs. Green: EGFP^+^ Tregs. Yellow: Treg tracks. Scale bar: 50 μm. (**B**) Treg track speed (μm/min). (**C**) Track straightness of single Tregs. Data represent the values of single cells in 3 individual mice per group (median ± IQR). Median values from CTLs 8, 10, and 14 dpi (shown in Figure 2) are indicated by the dashed lines. *P* values were obtained by Kruskal-Wallis test followed by Dunn’s multiple-comparison test. *P < 0.05.

Previous data indicated a low speed of Tregs in experimental autoimmune encephalomyelitis compared with the CTL speed we measured in the FV model (25). However, the general speed of Tregs largely varied within and between disease models (25, 26). The study of Treg motility is of great interest because it could influence the efficiency of Tregs to inhibit CTLs by enhancing the likelihood of potential CTL encounters (Figure 3A).

Therefore, we analyzed the motility of Tregs by single-cell tracking of EGFP^+^ Tregs in our intravital imaging videos in nondepleted DEREG mice. The expression of EGFP on the cell surface of Tregs in these mice enabled us to visualize them in the 2-photon experiments. However, the surface fluorescence signal remained low compared with the cytoplasmic expression of tdTom in our CTLs; this resulted in a smaller appearance of Tregs. We specifically looked at the Treg track speed and straightness, as these parameters were prominently changed in CTLs during FV infection (Figure 2). However, as opposed to CTLs, Treg motility only changed marginally during the course of FV infection, and Treg track speed showed no significant differences between the time points (Figure 3B). Also, Treg track straightness did not change between 8 dpi and 10 dpi, but showed a significant decrease at 14 dpi compared with 8 dpi (Figure 3C). We were curious to place the motility of Tregs in the context of the observed motility of CTLs, so we compared Treg motility parameters to the ones measured in CTLs. Interestingly, we found Tregs to be moving much more slowly compared with CTLs at all time points. The differences were most prominent at 10 dpi, where CTLs were 3 times faster than Tregs (Figure 3B and Supplemental Figure 1). Furthermore, Tregs showed higher track straightness at 10 dpi and lower track straightness at 14 dpi compared with CTLs (Figure 3C and Supplemental Figure 1B). Supplemental Figure 1 shows the data from Figure 3 in a statistical comparison between the CTL and Treg speeds and straightnesses of the same time point. These findings illustrate the highly motile behavior of CTLs during retrovirus infection, whereas Tregs only show limited motility. Thus, changes in Treg motility were not associated with the observed Treg-mediated CTL motility changes.

### Tregs have direct contacts with CTLs in vivo.

**Figure 4:**
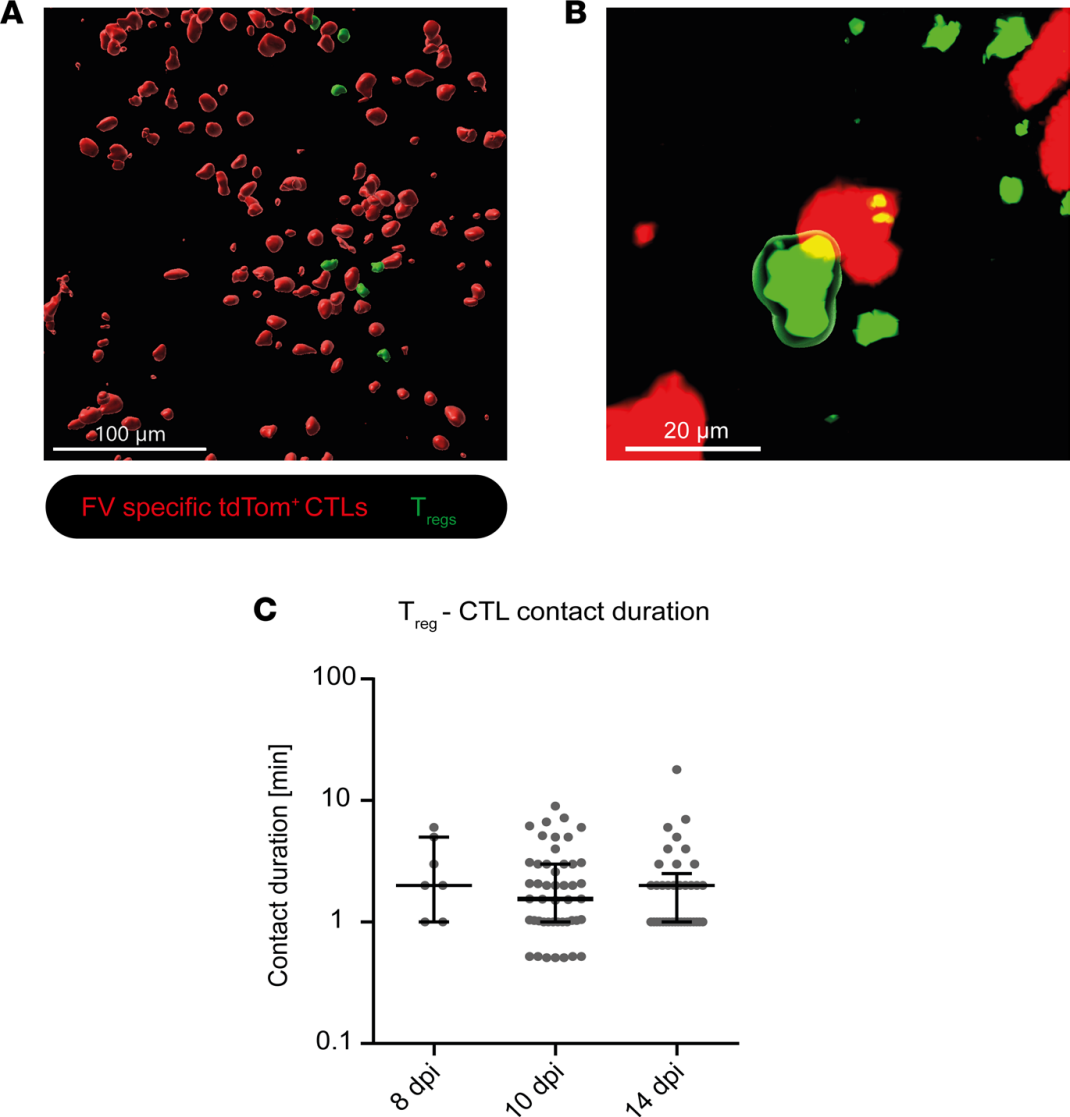
Direct interaction of Tregs with CTLs. Intravital 2-photon microscopy in the bone marrow: Contact between Tregs and FV-specific tdTom^+^ CTLs was analyzed in FV-infected, CTL-transferred DEREG mice 8, 10, and 14 dpi without Treg depletion. (**A**) Representative image of CTLs and Tregs with 3D surface rendering. Scale bar: 100 μm. (**B**) Representative contact of a Treg with a CTL. Contact is indicated by green surrounding the Treg. Track length indicates contact duration and was formed from the moment of contact until contact disruption. Scale bar: 20 μm. For the full movie, see Supplemental Video 2. Red: FV-specific tdTom^+^ CTLs. Green: EGFP^+^ Tregs. (**C**) Contact duration between Tregs and CTLs in minutes in 3 individual mice per group using intravital 2-photon microscopy in the bone marrow (median ± IQR). Numbers of contacts between Tregs and CTLs can be derived from the individual data points in the graph. *P* values were obtained by Kruskal-Wallis test followed by Dunn’s multiple-comparison test. *P < 0.05.

Tregs have a prominent impact on CTL motility in vivo and previous studies in the FV model showed that the Treg-mediated suppression of CTL responses in vitro was contact dependent (27). Based on these findings, we next investigated direct CTL-Treg contacts in vivo. One important parameter that increases the probability of cell-to-cell contact is the relative abundance of these cells. We quantified the frequencies of activated CD43^+^CD8^+^ T cells and Tregs in FV-infected mice at our selected time points of FV infection by flow cytometry (Supplemental Figure 2, A and B). We found low frequencies of Foxp3^+^CD4^+^ Tregs at 8 dpi, which only slightly increased during the course of infection in the bone marrow. However, the frequencies of CD43^+^CD8^+^ T cells were significantly increased at 10 and 14 dpi as compared with 8 dpi (Supplemental Figure 2, A and B). As increased numbers of CD43^+^CD8^+^ T cells and Tregs at 14 dpi might augment the likelihood of direct contacts between those 2 cell types, we analyzed CTL-Treg contacts at different time points after FV infection in vivo (Figure 4, A and B). Direct contacts between CTLs and Tregs were defined as a distance of CTLs to Tregs between 0 and 3 μm based on the preprocessing of the analyzed videos. Figure 4B shows a still image from a representative movie (Supplemental Video 2) in which contacts between CTLs and Tregs can be observed.

Investigating the number and duration of contacts between Tregs and CTLs, we observed increasing numbers of contacts at 10 and 14 dpi compared with 8 dpi (Figure 4C). This was likely due to the increase in CTL and Treg numbers at these time points compared with 8 dpi where only marginal numbers of T cells could be found in the bone marrow (15). To determine whether contact times between cells might influence the outcome, we additionally checked the contact duration between Tregs and CTLs. The contact times remained unchanged (1–4 minutes) at all observed time points (Figure 4C). Therefore, changes in CTL-Treg contact duration were not associated with the beginning CTL dysfunction at 14 dpi.

It was previously shown that Tregs and CTLs can express both programmed cell death protein 1 (PD-1) and programmed cell death ligand 1 (PD-L1) (28), and it has previously been shown that the PD-1/PD-L1 axis plays a role in the motility of T cells in LCMV infection (10). The importance of the PD-1/PD-L1 axis for CTL exhaustion has also been highlighted in the FV model (16, 29, 30). As interaction of Tregs and CTLs might involve PD-1 and PD-L1, we investigated the expression of both molecules on activated CTLs and Tregs at 14 dpi. We indeed found PD-1 and PD-L1 expression on both CTLs and Tregs, and activated CTLs showed a higher frequency of PD-1 expression compared with Tregs. Nearly all activated CTLs and Tregs were positive for PD-L1 (Supplemental Figure 2C). Therefore, the PD-1/PD-L1 axis might play a role in Treg-mediated inhibition of CTL motility.

### Active CTLs have only short contacts with their specific target cells in vivo.

**Figure 5:**
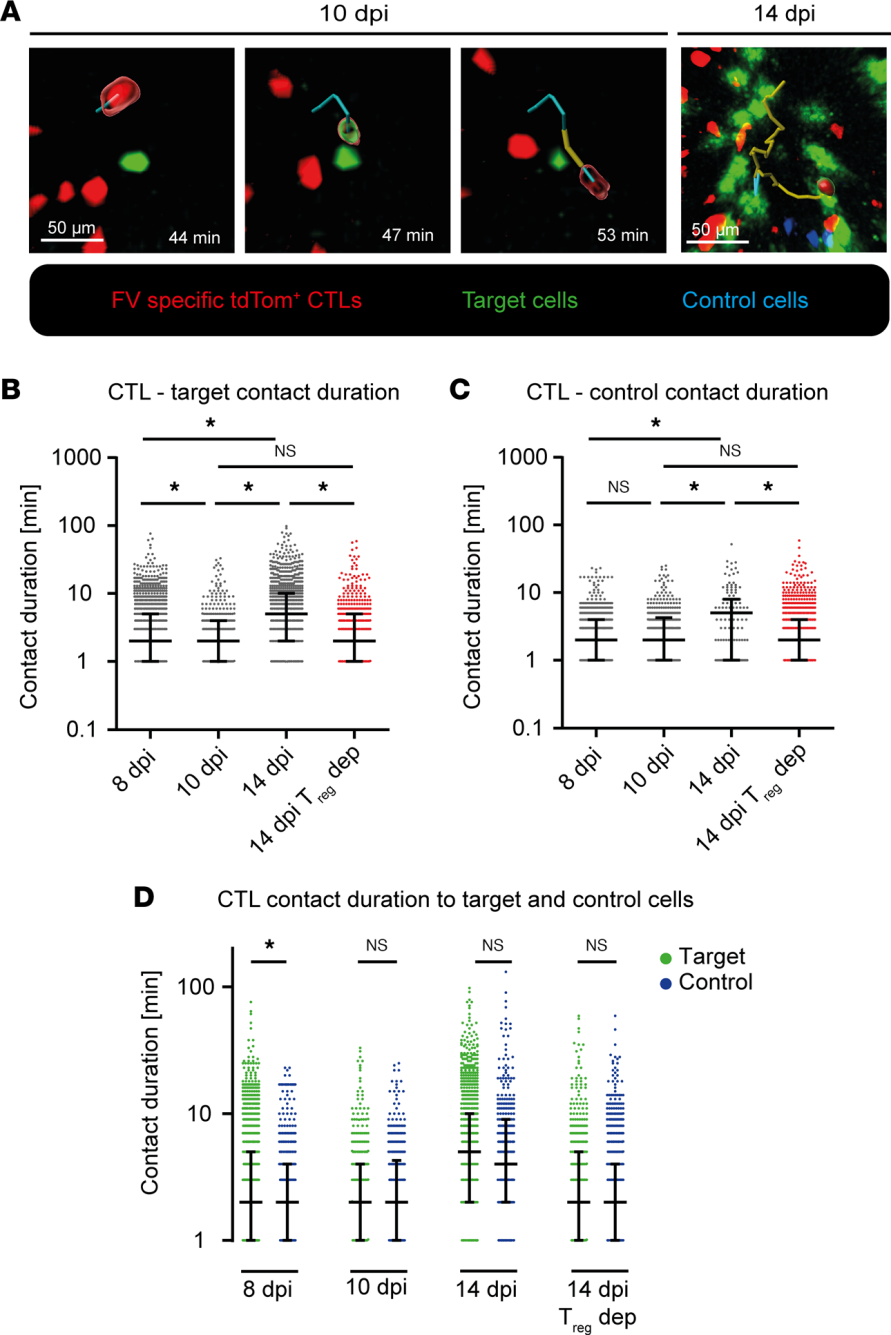
Contact with CTL targets. Intravital 2-photon microscopy in the bone marrow: FV-infected, CTL-transferred C57BL/6 or DEREG mice (for Treg depletion) were inoculated with a 1:1 mix of target and control cells 5 minutes before bone marrow imaging. Contact with target or control cells was assessed based on a distance of less than 3 μm. Contact duration was determined through automated CTL track generation from the moment of contact until contact disruption. (**A**) Representative contact between CTLs and targets 10 dpi and 14 dpi. Contact with target at 10 dpi is shown in a time series. Contact is indicated by green surrounding a CTL, and no contact by red surrounding a CTL. Red: FV-specific tdTom^+^ CTL. Green: target cell. Blue: control cell. Yellow track: CTL track without target cell contact. Blue track: CTL track with target cell contact. Scale bars: 50 μm. For full movies, see Supplemental Video 3 (10 dpi) and Supplemental Video 4 (14 dpi). (**B**) Contact duration of CTLs and target cells, in minutes. (**C**) Contact duration of CTLs and unloaded control cells, in minutes. (**D**) Comparison of CTL contact duration between contact with targets and control cells as shown in panels **A** and **B**. Data represent the values of individual cells in 3 individual mice per group (median ± IQR). *P* values were obtained by Kruskal-Wallis test followed by Dunn’s multiple-comparison test. *P < 0.05.

To observe contacts of CTLs with targets, we cotransferred into FV-infected mice a mixture of target and control cells differentially labeled with fluorescent dyes (Figure 1B, step 2). As target cells, we used primary B cells pulsed with FV peptide GagL^85–93^, which is the main immunogenic MHC class I epitope recognized by the transferred TCR-transgenic CD8^+^ T cells (31). We additionally transferred non–peptide-pulsed control cells stained in a different color to provide a comparison for the contact duration. Experiments without depletion of Tregs were carried out in C57BL/6 wild-type mice instead of DEREG mice to avoid the fluorescent signal of Tregs, as this detection channel was required for the visualization of the target cells. In one experimental group, DEREG mice were depleted of Tregs as described previously (20) to investigate their impact on the contact behavior of CTLs. Contacts with potential targets were identified based on a distance between 0 and 3 μm between tdTom^+^ CTLs and target or control cells in intravital imaging videos. Figure 5A shows representative CTL contacts with target cells.

We observed short contacts with targets at 8 dpi and even shorter contacts at 10 dpi (Figure 5, A and B, and Supplemental Video 3). This contrasted with significantly increased numbers of long contacts at 14 dpi (Figure 5, A and B, and Supplemental Video 4). When Tregs were depleted, CTLs at 14 dpi showed contact durations similar to undepleted mice at 10 dpi (Figure 5B). Highly motile CTL behavior at 10 dpi or after Treg depletion at 14 dpi (Figure 2, B–E) correlated with high cytotoxic capacity (15, 16), indicating that CTLs required only short contacts to scan and eliminate target cells at the peak of their response. High motility allowed multiple contacts with targets in a short period of time. Interestingly, the observed contact duration patterns of CTLs with peptide-loaded target cells were almost identical to those with nonspecific control cells (Figure 5C). Only at 8 dpi we observed shorter contact duration with control cells compared with the target cells (Figure 5D). Thus, the contact behavior of CTLs with target cells varied at different stages of acute FV infection and appeared independent of specific TCR recognition. Most importantly, these results also indicated that Tregs influenced both the biophysical interaction of CTLs with their targets and the overall motility of CTLs.

### Visualization of target cell killing by CTLs in FV-infected mice.

**Figure 6:**
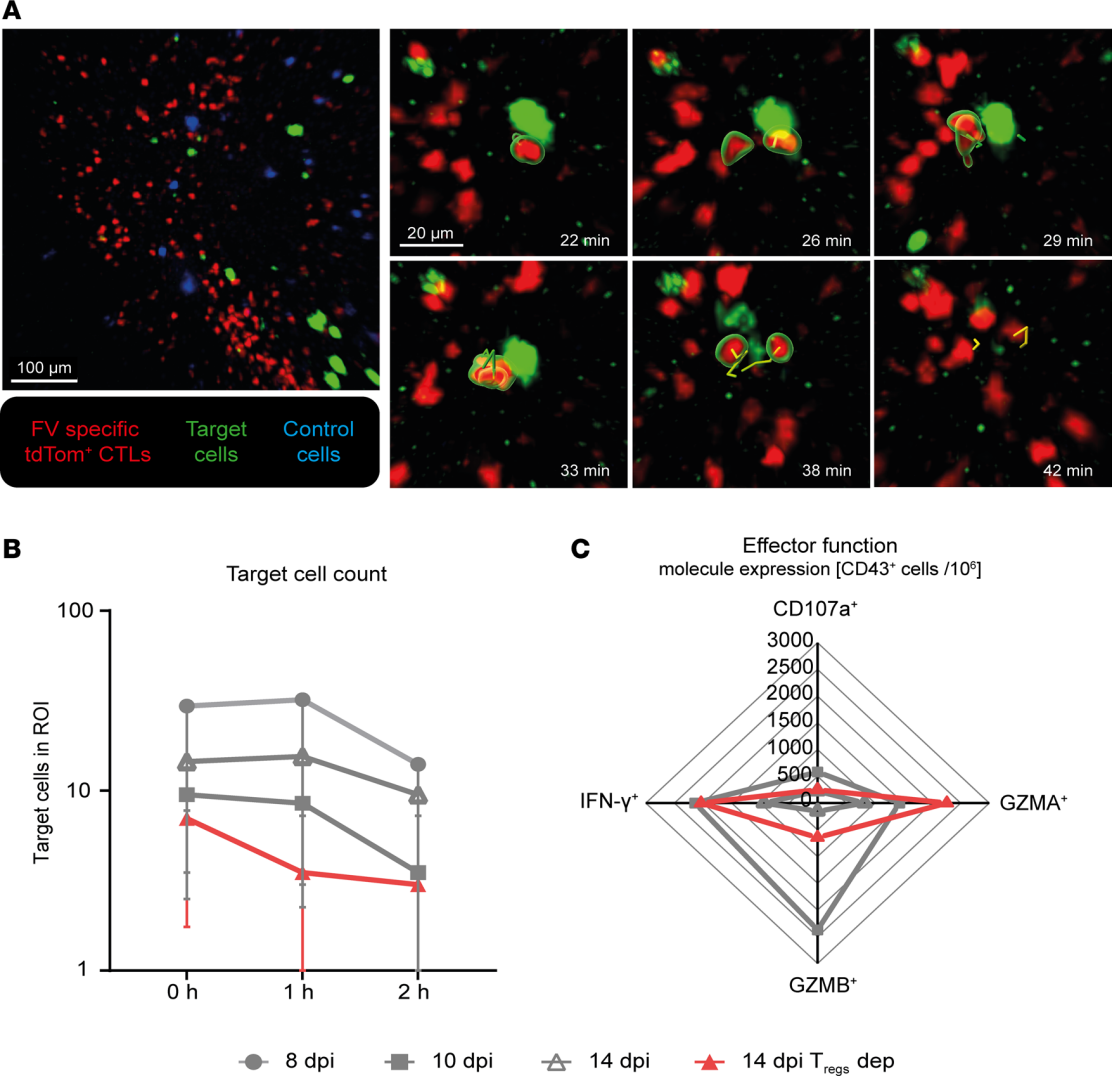
Killing of target cells. Intravital 2-photon microscopy in the bone marrow: Killing was determined in FV-infected, CTL-transferred C57BL/6 or DEREG mice (for Treg depletion) at 8, 10, and 14 dpi with and without Treg depletion. Target cell death was assessed based on fading of fluorescence and loss of clear cell structure. (**A**) Representative killing event caused by multiple CTL contacts 10 dpi in a time series. Contact was assessed based on a distance of less than 3 μm between an FV-specific tdTom^+^ CTL and target and highlighted by green surrounding the contacting CTL. The contact duration is indicated by the length of the track. Red: FV-specific tdTom^+^ CTLs. Green: target cells. Blue: control cells. Yellow track: CTL track while in contact with target cell. Scale bars: 100 μm (overview image) and 20 μm (zoomed-in images). For the full movie, see Supplemental Video 5. (**B**) Number of target cells over imaging time. Target count was evaluated at the beginning and at 1 and 2 hours of imaging. Data represent the median ± IQR of 6 individual mice per group. (**C**) Expression of cytotoxicity-associated molecules. Flow cytometry was used to identify the intracellular expression of GZMB, GZMA, and IFN-γ and the surface expression of CD107a on activated CD43^+^ CTLs in FV-infected mice 10 dpi and 14 dpi with and without Treg depletion. Radar chart represents the mean number of GZMB^+^, GZMA^+^, IFN-γ^+^, or CD107^+^CD43^+^ CTLs per million. The mean value was calculated from 1 experiment with 4 mice per group.

CTL contacts with targets do not automatically lead to killing and the duration of the contacts does not allow predictions about the efficiency of target cell elimination, the most important function of CTLs in virus infections. Previous publications from our group showed an increased CTL killing capacity at 10 and 14 dpi compared with 8 days after FV infection (15). Hence, we decided to directly visualize killing events through our intravital imaging approach. Killing events were indicated by the fading of target cell fluorescence and loss of a clear cellular structure. By these parameters, multiple killing events were observable in the intravital videos (Figure 6A and Supplemental Video 5). Frequently, multiple short contacts were visible before target cell death, confirming data from studies in murine CMV (mCMV) (5). However, we observed killing events after multiple visible CTL contacts and also after single observable contacts (Supplemental Video 5). We want to highlight that in our experimental setup, it was impossible to measure total numbers of contacts required for target cell elimination, as we could only observe the transferred fluorescent CTLs, but not the colorless endogenous CTLs. To obtain insights into the killing efficiency of CTLs, we quantified the target cell counts during the observation period in the imaged region of interest (ROI) as a correlate for target cell elimination. While we did not find a decrease in target cells at 8 dpi or 14 dpi, an efficient eradication of such targets was observed at 10 dpi. On 14 dpi, efficient target cell killing was only observed after Treg depletion (Figure 6B). For the 10-dpi group and 14-dpi Treg-depleted group, mostly short contact durations but high CTL motility were detected (Figures 2 and 5). To evaluate the molecular mechanisms underlying the loss of the cytotoxic capacity of CTLs at the beginning of T cell exhaustion (14 dpi) and the impact of Tregs on this phenomenon, we stained for granzyme B (GZMB), granzyme A (GZMA), and interferon γ (IFN-γ) in bone marrow CTLs from FV-infected mice at 10 and 14 dpi with and without depletion of Tregs. Cytotoxic granule release was characterized through surface staining for CD107a. The effector phenotype of CTLs was defined through surface staining for CD43. At 14 dpi, we detected a clear reduction in the count of GZMB^+^, GZMA^+^, and IFN-γ^+^ effector CTLs compared with 10 dpi, correlating with the onset of CTL exhaustion and reduced CTL motility. This effect was at least partially mediated by Tregs, as their depletion reverted it in part (Figure 6C). These data highlight that the time points when CTLs have short contact durations and efficient target cell elimination are associated with a highly cytotoxic phenotype. Furthermore., low expression of cytotoxic molecules was linked to low motility and target cell elimination.

To conclude, high CTL motility and cytotoxic molecule expression but only short contacts are required to efficiently eliminate target cells during acute FV infection. The beginning of CTL exhaustion is associated with a decrease in CTL motility, increased contact duration with targets, and a decrease in cytotoxic molecule expression, which altogether decrease efficient target cell elimination. During CTL exhaustion, Tregs show frequent direct contacts with CTLs and significantly regulate all these different features, including motility, target contact duration, and cytotoxic capacity in vivo.

## Discussion

In the present study, we visualized the motility of CTLs during the course of FV infection in which Tregs are known to suppress CTL activity and contribute to virus persistence (12, 19). We began our studies with the 8 dpi time point because 8 dpi has been shown to be the beginning of CTL responses with low numbers of FV-specific CTLs in the bone marrow, only moderate expression of cytotoxic effector molecules, and moderate CTL killing activity (15). At 8 dpi, we observed low CTL motility (Figure 2). The CTL response in FV infection has been shown to peak at 10 dpi, with high numbers of FV-specific CTLs and very efficient target cell killing (15). Interestingly, at this time point CTLs showed their highest track speed, track speed variation, and the longest track length and reduced track straightness (Figure 2, B–E). These results suggest that an efficient antiviral killer is a non-straight but fast-moving CTL that covers large areas in its search for targets. At 10 dpi, the median CTL track speed of around 8 μm/min (Figure 2B) was of the same general magnitude as the CTL speed observed in other in vivo and in vitro studies (5, 32–35). It has been shown that CTL speed can be influenced by various factors, including the disease model, vascular densities at CTL effector sites, and CTL activation status (5, 32–35). At 14 dpi, Treg responses peak in FV infections (8, 15), enabling us to study the influence of activated and expanded Tregs on CTLs in vivo. The presence of FV-induced Tregs was associated with reduced CTL speed and speed variation as well as increased track straightness, indicating that Tregs affected motility features that we found to be associated with an effective cytotoxic CD8^+^ T cell. Our Treg depletion experiments demonstrate that the impaired CTL motility during the late phase of acute FV infection is mediated to a large degree by Tregs. Depletion of Tregs aligned multiple motility parameters to the level of the peak CTL response at 10 dpi (Figure 2). Previous Treg depletion experiments demonstrated the restoration of cytotoxic molecules and IFN-γ as well as the cytotoxic activity of CTLs (12, 15, 36), which we were able to validate here for the late-acute phase of FV infection. The impairment of CTL motility and cytotoxic function results in incomplete clearance of virus-infected cells and the development of viral chronicity, which are at least partially reversible by Treg depletion (19).

The analysis of the CTL-target interactions by intravital imaging revealed that the changes in CTL motility influenced the responses toward FV-peptide-presenting target cells. At the peak of the CTL response at 10 dpi, we observed multiple short contacts between CTLs and target cells before their subsequent killing (Figure 6 and Supplemental Video 5). Our data are in accordance with published data from a mCMV mouse study, where on average 3.5 contacts provided by multiple CTLs were observed before target cell killing (5). In our study, the exact number of contacts between CTLs and targets prior to target cell elimination could not be determined because only the exogenously added FV-specific CTLs were fluorescent and the endogenous FV-specific CTLs were invisible in the intravital imaging. Individual contacts between CTLs and FV-loaded target cells were 4 times shorter compared with the contact durations reported for mCMV-infected cells by Halle et al. (5). Interestingly, contacts between CTLs and target cells were prolonged at 14 dpi, when very little target cell killing was observed (Figure 5B). Hence, long contacts were not necessarily associated with efficient target cell killing during FV infection. Long contacts might even deteriorate effective CTL responses, by reducing speed and preventing many short CTL-target cell contacts in a given period of time. In melanoma mouse models, contact durations of CTLs to tumor cells varied between minutes and several hours in vitro and longer contact durations were associated with increased sublethal hit delivery. In these studies, in vivo contacts were 7.5 times longer compared with what we describe here at the peak of the CTL response against FV (11). Moreover, the duration of CTL contacts with FV-labeled target cells, which could be specifically recognized by the TCR of the transgenic CTL, was almost indistinguishable from non–peptide-loaded control cells in our experiments (Figure 5, B and C). This contrasts with described tumor cell targets of CTL killing, where nonspecific contacts were between 3 and 12 times shorter compared with specific contacts. (11). Hence, CTL-target contacts in antitumor immunity appear to substantially differ from cytotoxic antiviral responses.

Interestingly, the depletion of Tregs also had an impact on the contact behavior of CTLs with target cells. Treg depletion at 14 dpi equalized the CTL–target cell contact duration to results from 10 dpi (Figure 5B), showing that, apart from CTL motility, Tregs also influence the CTL contact behavior with target cells in the course of acute FV infection. At 14 dpi, CTLs seemed to be less able to migrate to and detach from targets and subsequently inefficient in eliminating it. Depletion of Tregs led to higher motility and decreased contact duration at 14 dpi (Figures 2 and 5), restoring this important aspect of effective CTL responses in FV infection.

A possible mechanism for how Tregs inhibit CTL motility and their antiviral function might be through direct contact between Tregs and CTLs. In an in vitro study with FV-induced Tregs, Robertson et al. showed that Treg-mediated inhibition of cytotoxic molecules in CTLs required physical Treg-CTL contacts and did not rely on the presence of antigen-presenting cells (27). We were able to visualize several contacts between Tregs and CTLs in vivo (Figure 4 and Supplemental Video 2). Contacts between CTLs and Tregs have been previously shown to occur in lymph nodes by Mempel et al. (35) and were comparably short lived, as in our study. The contact duration, at a median of approximately 2 minutes, was very stable throughout the course of FV infection (Figure 4C) and leads us to the conclusion that the length of the CTL-Treg contact does not decide the inhibitory outcome. It is more likely that the inhibitory effect of Tregs is dependent on the pure abundance of Tregs and CTLs. In the late-acute phase of FV infection, large numbers of CTLs are present in the bone marrow due to infiltration and expansion (8, 15), which makes their contacts with Tregs more likely and frequent. Previous work by our group showed a high activation status of Tregs at 12 days after FV infection compared with Tregs from naive mice, characterized by expression of CD69, CD43, and CD103 (37). The high activation status of Tregs together with the increased probability of contacts between CTLs and Tregs might contribute to Treg-mediated inhibition of CTL motility in the late-acute phase of infection. In addition, we show here that activated Tregs improve their motility, which was reflected in a reduced track straightness (Figure 3C), a feature that might increase the likelihood of contacting other cells. This was observed at the peak of the Treg response in FV infection at 14 dpi.

Tregs are known to inhibit effector T cell functions by mechanisms including the expression of suppressive molecules such as PD-1, PD-L1, and CTL-associated protein 4 (CTLA-4), production of immunoregulatory cytokines, consumption of available interleukin 2, and transfer of suppressive molecules such as cAMP. Some mechanisms, such as CTLA-4–induced signaling and cAMP transfer, require direct contacts between Tregs and effector T cells (19, 28, 38, 39). Our current results add the modulation of CTL motility to the features that are regulated in effector T cells by Tregs. However, the molecular mechanism through which Tregs contact CTLs and inhibit their motility remains to be determined. Contacts between Tregs and conventional CD4^+^ T cells have been shown to inhibit CD4^+^ T cell activity via a gap junction–based cAMP transfer from the Tregs to the targets (38). In addition, Tregs have been demonstrated to control HIV replication in T cells through cAMP (40). Increased cAMP concentrations have been shown to impair CTL morphology and killing capacity in vitro (41). Additionally, the PD-1/PD-L1 axis has been shown to contribute to Treg-mediated suppression of CTL proliferation and functionality in LCMV-infected mice (28). Interestingly, blocking the PD-1/PD-L1 axis in LCMV infection also improved CTL motility (10), highlighting immune checkpoint molecules as potential molecular mediators of CTL motility. In the FV model, the PD-1/PD-L1 axis as well as Tregs regulate the CTL activity and contribute to CTL exhaustion. However, it is not fully clear whether these mechanisms overlap or are rather independent. A study in chronically FV-infected mice showed that combined blocking of Tregs and PD-1 had synergistic effects on CTL reactivation (29). This suggests that the PD-1/PD-L1 axis might not be the main molecular mechanism through which Tregs impair antiviral CTL responses, at least not in FV infection (29, 42). We assume that the motility of CTLs might be impaired by multiple molecular effectors. Possible mediators of CTL motility are chemokine receptors, such as CXCR3 (43, 44), but it was previously reported that the expression of chemokine receptors and their specific impact on cell motility was dependent on the infection model used (5). In our model, we did not find an increase in CXCR3 expression on CTLs during the course of FV infection (unpublished observation). Thus, the molecular mechanisms remain elusive, and identifying these mechanisms is very difficult in complex intravital imaging systems.

The applicability of the widely described phenomenon of “serial killing” to our model remains an open question. Serial killing has been extensively described in different disease models (11, 45). Previous studies and reviews have already discussed the importance of this phenomenon in in vivo studies during antiviral immune responses (5, 45). We were not able to evaluate how many targets were killed by a single CTL in our data set, due to technical limitations. It was previously described that serial killer CTLs only form a small subgroup of CTLs that eliminate virus-infected cells and that the described importance of this CTL function exaggerates the observable in vivo killing capacity (5, 45). However, our data support the notion that efficient target cell elimination by CTLs in vivo does not require long-lasting, stable cytotoxic synapses (5, 46) but rather fast moving CTLs with short contacts with targets.

To conclude, our study demonstrates the importance of CTL motility for the quality of antiretroviral CTL responses. Furthermore, we identified Tregs as important regulators of CTL motility, which can initiate T cell exhaustion and the development of chronic infection. CTL motility changes in vivo will likely be of relevance and interest in other infections and in cancer. The identification of the molecular program that changes CTL motility will likely lead to new therapeutic approaches for both enhancing CTL function in antiviral activity and regulating CTL activity in immunopathogenesis. Increasing the motility of CTLs could improve viral clearance and cancer elimination, which would be of great therapeutic interest in future medicine.

## Methods

### Mice.

Experiments were performed using 8- to 70-week-old female and male C57BL/6 (C57BL/6JOlaHsd, Envigo) and DEREG-transgenic C57BL/6 mice as recipient mice. DEREG mice were originally provided by Tim Sparwasser (current affiliation: Institute of Medical Microbiology and Hygiene, University Medical Center of the Johannes Gutenberg-University Mainz, Mainz, Germany) and bred at the University of Duisburg-Essen and University Hospital Essen under pathogen-free conditions. DEREG (H-2b) BAC–transgenic mice express a simian diphtheria toxin receptor–enhanced GFP (DTR-EGFP) fusion protein under control of the endogenous forkhead box P3 (*Foxp3*) promoter/enhancer regions on the BAC transgene (20). TCR-Lck-tdTom donor mice, containing more than 90% GagL^85–93^ epitope–specific tdTomato-expressing CTLs, were generated by 2 consecutive matings on a C57BL/6 background, as previously described by Otto et al. (22). As a first step, *dLck-hcre*–transgenic mice (stock number 012837, The Jackson Laboratory), expressing a *cre* recombinase gene under the control of the distal lymphocyte protein tyrosine kinase (*dLck*) promoter (47), were bred with ROSA26-*loxP*-Stop-*loxP*-tdTomato reporter mice (48) (stock number 007909, The Jackson Laboratory). This results in T cell–specific tdTomato expression. As the next breeding step, the offspring were crossed with D^b^ GagL TCR^tg^ mice that express an FV-specific TCR directed against the GagL^85–93^ epitope on more than 90% of all CD8^+^ T cells (49), and were originally provided by Philip D. Greenberg (Department of Immunology, University of Washington, Seattle, Washington, USA). All mice from these matings were bred at the University Hospital Essen under pathogen-free conditions. Donor mice were narcotized for approximately 30 seconds with 5 vol% isoflurane (Forene 100% [v/v], AbbVie) mixed with oxygen as carrier gas before blood draw. Blood was drawn via retrobulbar puncture with a sodium heparin–coated capillary (Hirschmann). Each donor mouse was used in time intervals of a minimum of 2 weeks between blood draws for recovery. A maximum of 5 blood draws were carried out per donor mouse.

### Virus and viral infection.

The used FV stock contained a complex of B-tropic Friend murine leukemia helper virus (F-MuLV) and spleen focus-forming virus. The virus stock was prepared as a 10% spleen cell homogenate from BALB/c mice infected for 14 days with 3000 spleen focus-forming units and noncloned virus stock (50). Recipient mice were infected by intravenous injection of 20,000 spleen focus-forming units of FV in 100 μL PBS. FV-mWasabi stocks were generated as previously described (21). The virus stocks were free of lactate dehydrogenase–elevating virus (51).

### Cell isolation and adoptive transfer.

From donor mice, approximately 100 μL of blood was collected in an EDTA-coated tube (Sarstedt). Erythrocytes were lysed in 5 mL of 0.16 M ammonium carbonate (pH 7.2) on ice with occasional shaking for 5 minutes, followed by washing with 35 mL PBS. CD8^+^ T cells were isolated by magnetic activated cell sorting using a mouse CD8a^+^ T Cell Isolation Kit (Miltenyi Biotec) following the manufacturer’s instructions. One thousand purified cells were transferred intravenously suspended in 100 μL PBS into recipient mice, which had been infected with FV 4 hours prior to the cell transfer.

### Depletion of Tregs.

For depletion of Tregs, the DEREG diphtheria toxin depletion model was used (20). Mice were injected with diphtheria toxin (Merck) diluted in PBS. A total of 0.5 μg was inoculated intraperitoneally 3 times (on days 7, 10, and 13) after FV infection.

### Preparation of target and control cells.

B cells were isolated from spleen homogenate of C57BL/6 mice by using a mouse Pan B Cell Isolation Kit II (130-104-443, Miltenyi Biotec) following the manufacturer’s instructions. Half of the isolated B cells were loaded with 2.5 μM D^b^GagL peptide (PanaTecs) (31) and stained with 1 μM Cell Trace CFSE (Invitrogen) (target cells). The other half of the isolated cells were left unpulsed and stained using a QTracker 655 cell staining kit (Invitrogen) (control cells). Cells (1.5 × 10^7^) of the cell mix or target cells (7.5 × 10^6^) were adoptively transferred to the recipient mice 5 minutes before the beginning of the bone marrow surgery required for the intravital imaging.

### Intravital 2-photon imaging.

Mice were injected with 100 mg/kg ketamine and 20 mg/kg xylazine diluted in 0.9% isotone sodium chloride solution prior to intubation and surgery and kept narcotized using 1%–2% isoflurane in O_2_ for the time of imaging. Intravital imaging and surgery were carried out as described previously (24, 52). After bone marrow surgery, the mouse was put into a 37°C warm NaCl bath, in which the bone marrow was imaged. Two-photon microscopy was done using a Leica TCS SP8 MP microscope with an HCX IRAPO L25×/0.95-NA water-immersion objective, 2 external hybrid reflected-light detectors (HyDs), and 2 external photomultiplier tubes (PMTs). Imaging was performed with a titanium-sapphire laser (Coherent Cameleon Vision II) tuned to 950 nm for intravital microscopy. FV-specific tdTomato^+^ CTLs (PMT, 585/40 filter), solid bone visualized by second-harmonic-generation (SHG) signal (HyD, 460/50 filter), CFSE-stained target cells (HyD, 525/50 filter), and QTracker 655–stained control cells (PMT 650/50 filter) or blood flow visualized through QTracker 655 (Invitrogen) were detected. For videos, one *z*-stack of 227.86 μm per minute or less time with a step size of 3 μm, an imaging speed of 400 Hz, and a pixel size of 1.16 was recorded in a 512 μm × 512 μm format for a collective video time of up to 3 hours. Videos were recorded using LAS X software (Leica Microsystems).

### Movie analysis.

LAS X files were converted with the IMARIS file converter (Bitplane) and preprocessed and analyzed in IMARIS 9 (Bitplane). For preprocessing, all channels were subtracted from each other to eliminate channel spillover and shifts in the videos were corrected with IMARIS shift correction if necessary. For the evaluation of cell motility parameters, videos of a length of approximately 30 minutes were analyzed. For automated tracking, a frame gap size of between 0 and 3 frames and a maximum distance of 20 μm between frames were chosen based on the video quality. Track speed, track speed variation (ratio of track standard deviation and tracks speed mean), and track straightness (ratio of direct track distance and track length) were determined through automated tracking of CTLs filtered for a minimum track duration of 5 minutes. For the analysis of track length in 10 minutes, the track length of cells with a track duration for a minimum of 10 minutes were filtered and analyzed. For investigation of cell-to-cell contact, the full length of the generated videos was used (approximately 3 hours). Surfaces were generated for the different cell populations and contacts were assessed based on a distance between 0 and 3 μm based on video quality. For quantification of cell count, the cells were automatically counted in each frame using the spot or surface function. Movies were excluded from analysis if no cell motility was observable.

### Flow cytometry.

Cell surface staining was performed using the following antibodies: anti-CD3e (BUV496, clone 145-2C11, catalog 612955, BD Biosciences), anti-CD107a (FITC, clone 1D4B, catalog 121606, BioLegend), anti-CD43 (FITC, clone 1B11, catalog 121206; PE-Cy7, clone 1B11, catalog 121218; and PE/Dazzle 594, clone 1B11, catalog 121226; all BioLegend), anti-CD8a (BV605, clone 53-6.7, catalog 100744, BioLegend and BUV805, clone 53-6.7, catalog 612898, BD Biosciences), anti-CD4 (Alexa Fluor 700, clone RM4-5, catalog 557956; BV605, clone RM4-5, catalog 563151; BUV563, clone GK1.5, catalog 612923; all BD Biosciences), anti-CD25 (PerCP, clone PC61, catalog 102028 and BV785, clone PC61, catalog 102051, BioLegend), anti–PD-1 (APC, clone 29F.1A12, catalog 135210, BioLegend), and anti–PD-L1 (PE, clone MIH5, catalog 12-5982-82, eBioscience). To exclude dead cells from analysis, the fixable viability dye (eF780, eBioscience), Zombie Aqua Fixable Viability Kit (BioLegend), and 7-aminoactinomycin D (BD Biosciences) were used for the exclusion of dead cells. To maintain the cytoplasmic tdTomato signal, cells were prefixed (3.5 minutes) using the Cytofix/Cytoperm kit (BD Biosciences) as described previously (53). For intracellular staining, a second fixation/permeabilization using the Cytofix/Cytoperm kit (BD Biosciences) for a minimum of 30 minutes was performed. The following antibodies were used for intracellular staining: anti-perforin (APC, clone S16009B, catalog 154404, BioLegend), anti-GZMB (BB790-P, clone GB11, catalog 624296, BD Biosciences), anti-GZMA (eF450, clone GzA-3G8.5, catalog 48-5831-82, Invitrogen), and anti–IFN-γ (FITC, clone XGM1.2, catalog 11-7311-41, eBioscience). For intranuclear staining of Foxp3, the Foxp3/Transcription Factor Staining Buffer Set (eBioscience) was used together with anti-Foxp3 antibody (APC, clone FJK-16s, catalog 17-5773-82 and FITC, clone FJK-16s, catalog 11-5773-82, eBioscience). For intracellular IFN-γ staining, cells were first stimulated for 1 hour at 37°C using 25 ng/mL phorbol myristate acetate (Sigma-Aldrich) and 0.5 μg/mL ionomycin (Enzo) in RPMI medium, followed by 2-hour incubation after addition of 1× monensin (BioLegend) and 2 μg/mL Brefeldin A (Sigma-Aldrich) in RPMI medium. Samples were acquired on a BD Symphony A5 cytometer or LSR II flow cytometer and a total of 200,000 to 1,000,000 events were recorded. Analyses were performed using the FACSDiva and FlowJo 10.7 software (BD Biosciences).

### Statistics.

GraphPad Prism version 8 software was used for statistical analyses. To determine statistical significance between multiple groups, Kruskal-Wallis 1-way ANOVA on ranks with Dunn’s multiple-comparison test was used. Differences were defined to be significant from *P* values of less than 0.05. Radar charts were created with OriginPro 9.0 (OriginLab Corporation). Adobe Illustrator CC 2018, Microsoft Powerpoint, and Adobe Premiere Pro CC 2018 were used for figure and movie design.

### Study approval.

All animal experiments were conducted in accordance with the regulations of the local animal welfare regulations and reviewed by the central animal laboratory (ZTL) and office for nature, environment, and consumer protection of North-Rhine Westphalia (LANUV). Mice were kept in pathogen-free conditions and handled in accordance with institutional guidelines.

### Data availability.

Supporting numerical data values presented as graphs are uploaded as supplemental material. Raw ims files generated by 2-photon microcopy and fcs files generated by flow cytometry are available upon request from the corresponding author.

## Author contributions

MG and UD designed the study. DM and LO conducted the experiments with the help of JW, ZL, AK, and AB. DM performed final data analysis. AH and GZ contributed to new methods. DM wrote the original draft of the manuscript under the guidance and revision of UD, MG, and AH. KTH edited the manuscript. All authors reviewed and edited the manuscript.

## Supplementary Material

Supplemental data

Supplemental video 1

Supplemental video 2

Supplemental video 3

Supplemental video 4

Supplemental video 5

Supporting data values

## Version 1 07/10/2023

Electronic publication
