# Supplementary material for: Regulatory T cells suppress the motility of cytotoxic T cells in Friend retrovirus–infected mice
Source: JCI Insight. 2023 Jul 10;8(13):e167482. doi: 10.1172/jci.insight.167482 (PMC10371334; doi:10.1172/jci.insight.167482)
Supplement: Supplemental data [file jciinsight-8-167482-s118.pdf]

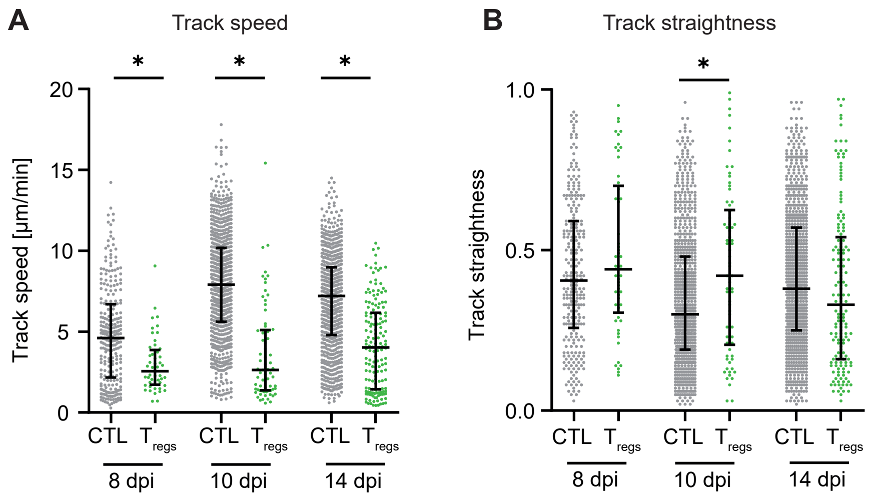

Supplementary Figure 1: T_{reg} motility compared to CTL motility. Intravital two-photon microscopy: T_{reg} and CTL motility was assessed in two-photon intravital imaging videos in the tibial bone. Different statistical comparison of data previously shown in figure 2 and 3. **(A)** T_{reg} speed and **(B)** T_{reg} track straightness compared to the respective parameters in CTL motility. Data represents the values of single cells in 3 individual mice per group (median±IQR). P-values were obtained by Kruskal Wallis test followed by corrected Dunn's multiple comparison test. * $p<0.05$.

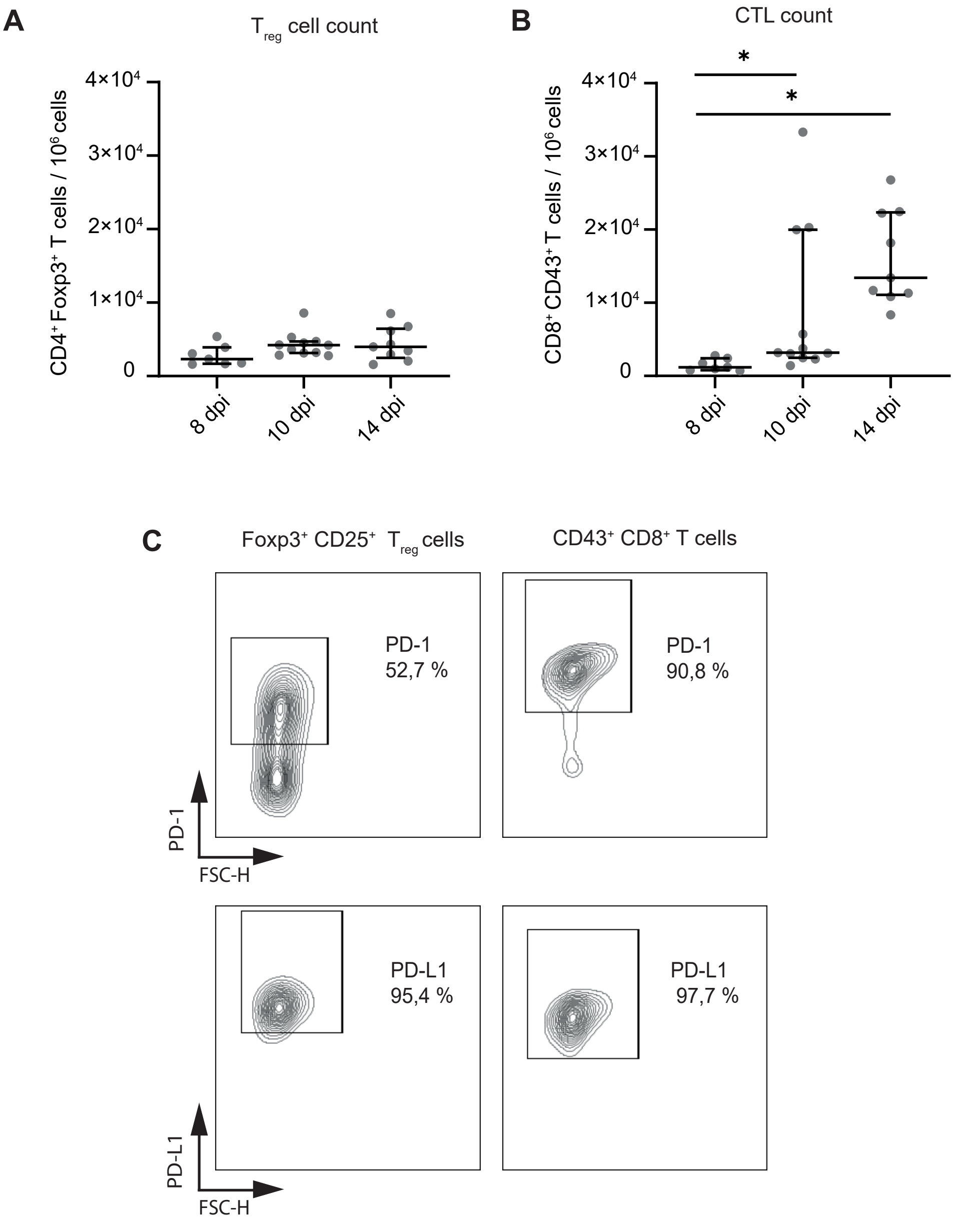

Supplementary Figure 2: CTL and T_{reg} frequencies. Frequencies of **(A)** Foxp3$^+$ T_{reg} cells and **(B)** CD43$^+$ CD8$^+$ T cells per 10^6 bone marrow cells measured by flow cytometry in 7-11 mice in 2-3 separate experiments (median±IQR). P-values were obtained by Kruskal Wallis test followed by corrected Dunn's multiple comparison test. * $p<0.05$. **(C)** Representative contour plots of PD-1 and PD-L1 expression on Foxp3$^+$ CD25$^+$ T_{reg} and activated CD43$^+$ CD8$^+$ T cells in the bone marrow of mice 14 days post FV infection. Experiment was carried out one time in 2 mice.

Supplementary Movie 1: Evaluation of CTL motility. Intravital two-photon microscopy in the bone marrow: representative video of CTL tracking. Video was generated in FV-infected CTL transferred mouse 14 dpi. Red: FV-specific $tdTom^+$ CTLs, yellow tracks: CTL tracks, grey: tibial bone (SHG). Frame frequency: 5 fps. Tracks were generated automatically in IMARIS 9. A Snapshot of the video is included in figure 1.

Supplementary Movie 2: T_{regs} form direct contacts to CTLs. Intravital two-photon microscopy in the bone marrow: representative video of T_{reg}-CTL contact. Video generated in FV-infected CTL-transferred mouse 14 dpi; red: CTL, green: $EGFP^+$ T_{reg}, yellow track: T_{reg} track while in contact with CTL. Frame frequency: 5 fps. Contact was assessed based on a distance of < 3 µm between T_{reg} and CTL and highlighted through green surrounding of the contacting T_{regs}. From the moment of contact until contact disruption T_{regs} were automatically tracked to determine contact duration. Exemplary picture of the T_{reg}-CTL-contact is incorporated in Figure 4.

Supplementary Movie 3: Short CTL contact 10 days post FV infection. Intravital two-photon microscopy in the bone marrow: exemplary video of CTL-target cell contacts 10 dpi. Frame frequency: 5 fps. Contact was assessed based on a distance of < 3 µm between FV-specific $tdTom^+$ CTL and target and highlighted through green surrounding of the contacting CTL. From the moment of contact until contact disruption CTLs were automatically tracked to determine contact duration. Red: CTL, green: target cell, blue track: CTL track when no contact, yellow track: CTL track when in contact to target. Snapshots of the video are included in figure 5.

Supplementary Movie 4: Long CTL contact 14 days post FV infection. Intravital two-photon microscopy in the bone marrow: exemplary video of CTL-target cell contacts 14 dpi. Frame frequency: 5 fps. Contact was assessed based on a distance of < 3 µm between FV-specific tdTom^{+} CTL and target and highlighted through green surrounding of the contacting CTL. From the moment of contact until contact disruption CTLs were automatically tracked to determine contact duration. Red: CTL, green: target, blue track: CTL track when no contact, yellow track: CTL track when in contact to target. Tracks were generated to display full track length. The moment of contact disruption is indicated by loss of green surrounding of the CTL. A Snapshot of the video is included in figure 5.

Supplementary Movie 5: Multiple short CTL contacts lead to target cell elimination. Intravital two-photon microscopy in the bone marrow: representative video of killing event caused by multiple CTL contacts 10 dpi. Frame frequency: 7.5 fps, slow motion when target cell fades: 1,75 fps. Contact was assessed based on a distance of < 3 µm between FV-specific tdTom^{+} CTL and target cell and highlighted through green surrounding of the contacting CTL. The contact duration is indicated by the length of the track. Red: FV-specific tdTom^{+} CTLs, green: target cells, blue: control cells, yellow track: CTL track while in contact to target cell. Snapshots of the video are included in figure 6.
